# Predicting the targets of IRF8 and NFATc1 during osteoclast differentiation using the machine learning method framework cTAP

**DOI:** 10.1186/s12864-021-08159-z

**Published:** 2022-01-07

**Authors:** Honglin Wang, Pujan Joshi, Seung-Hyun Hong, Peter F. Maye, David W. Rowe, Dong-Guk Shin

**Affiliations:** 1 Computer Science and Engineering Department, University of Connecticut, Storrs, USA; 2 Department of Reconstructive Sciences, University of Connecticut Health Center, Farmington, USA; 3 Center for Regenerative Medicine and Skeletal Development, University of Connecticut Health Center, Farmington, USA

**Keywords:** Osteoclast differentiation, IRF8, NFATc1, Target prediction, Machine learning

## Abstract

**Background:**

Interferon regulatory factor-8 (IRF8) and nuclear factor-activated T cells c1 (NFATc1) are two transcription factors that have an important role in osteoclast differentiation. Thanks to ChIP-seq technology, scientists can now estimate potential genome-wide target genes of IRF8 and NFATc1. However, finding target genes that are consistently up-regulated or down-regulated across different studies is hard because it requires analysis of a large number of high-throughput expression studies from a comparable context.

**Method:**

We have developed a machine learning based method, called, Cohort-based TF target prediction system (cTAP) to overcome this problem. This method assumes that the pathway involving the transcription factors of interest is featured with multiple “functional groups” of marker genes pertaining to the concerned biological process. It uses two notions, Gene-Present Sufficiently (GP) and Gene-Absent Insufficiently (GA), in addition to log2 fold changes of differentially expressed genes for the prediction. Target prediction is made by applying multiple machine-learning models, which learn the patterns of GP and GA from log2 fold changes and four types of Z scores from the normalized cohort’s gene expression data. The learned patterns are then associated with the putative transcription factor targets to identify genes that consistently exhibit Up/Down gene regulation patterns within the cohort. We applied this method to 11 publicly available GEO data sets related to osteoclastgenesis.

**Result:**

Our experiment identified a small number of Up/Down IRF8 and NFATc1 target genes as relevant to osteoclast differentiation. The machine learning models using GP and GA produced NFATc1 and IRF8 target genes different than simply using a log2 fold change alone. Our literature survey revealed that all predicted target genes have known roles in bone remodeling, specifically related to the immune system and osteoclast formation and functions, suggesting confidence and validity in our method.

**Conclusion:**

cTAP was motivated by recognizing that biologists tend to use Z score values present in data sets for the analysis. However, using cTAP effectively presupposes assembling a sizable cohort of gene expression data sets within a comparable context. As public gene expression data repositories grow, the need to use cohort-based analysis method like cTAP will become increasingly important.

## Background

Bone is a dynamic organ constantly being remodeled to maintain its strength and quality. During remodeling, old bone is removed by osteoclasts and new bone is generated by osteoblasts. Bone homeostasis is the delicate balance between levels of bone resorption and bone formation. Bone diseases like osteoporosis and periodontitis are notoriously due to enhanced bone resorption from increased osteoclast differentiation. To explore a gene’s behavior under this context, two important transcription factors (TFs), Interferon regulatory factor-8 (IRF8) and nuclear factor-activated T cells c1 (NFATc1), have been extensively studied [1–3]. IRF8, a transcription factor expressed in immune cells, is a key regulatory molecule in suppressing osteoclastogenesis [1]. In contrast, NFATc1 functions as a master transcriptional regulator of osteoclast differentiation [2]. As reported in [3], IRF8 directly interacts with NFATc1 to prevent NFATc1’s translocation into the nucleus. Although the roles of IRF8 and NFATc1 are known, what target genes mediate the functional outcomes of these two TFs is not well established. Luckily, ENCODE has generated chromatin immunoprecipitation sequencing (ChIP-seq) data for both IRF8 and NFATc1, which has enabled us to investigate their possible direct target genes.

In BioTarget [4], we demonstrated how targets of TBX21 and GATA2 can be identified, respectively, for the differentiation of Th1 and Th2 immune cells by analyzing the respective ENCODE ChIP-Seq data over five different TCGA cancer cohort data sets, Stomach Adenocarcinoma (STAD), Breast Invasive Carcinoma (BRCA), Colon Adenocarcinoma (COAD), Lung Adenocarcinoma (LUAD), and Lung Squamous Cell Carcinoma (LUSC). This work reported that TFs may share some common target genes across different cancer types, but they also have unique target genes. This finding is not unexpected since it has been postulated that depending on the temporal and spatial context of a biological system, the role of TF’s may change and it could use different genes to undertake its programmed functions.

Predicting gene targets of a TF is an effort to extend existing biological knowledge which has been typically curated in the form of signal transduction pathways, molecular pathways or gene regulatory networks. In this regard, BioTarget and cTAP are efforts to extend known pathways through an integrative analysis of combining ChIP-seq data with many gene expression data sets from experiments of comparable context. experiments. The system we are describing in this work is called Cohort-based TF target prediction system (cTAP) and it is an attempt to extend our previous work BioTarget in multiple ways. First, cTAP uses different pathway components in computing TF gene target prediction. While BioTarget uses pathway routes for prediction, cTAP uses “functional groups” which are embedded in the pathway to predict the TFs’ target genes. Second, cTAP tackles the problem of using data from different laboratories to build a cohort of comparable data sets unlike BioTarget’s use of an established cohort data set like TCGA. Third, cTAP introduces a new idea of using Z scores of intensity values of gene expression, in addition to log2 fold changes (log2FCs) of test vs. control gene expression values. Fourth, machine-learning methods are used to predict gene regulation instead of BioTarget’s algorithmic way of using log2FCs. Finally, two new measures, called, Gene-Present Sufficiently (GP) and Gene-Absent Insufficiently (GA), are computed and these measures are used in the final stage of “adjusting” target prediction.

Machine-learning approaches have been applied in bioinformatics field for decades [5–9]. Hasan et al. proposed machine learning based models that use support vector machine (SVM), logistic regression (LR) and Naïve Bayes (NB) etc. to identify neuropeptide [10], and DNA *N*^6^-methyladenine sites of plant genomes [11]. In [12], Basith et al. developed a machine learning framework to identify cell-specific enhancers from the human genome. Computationally predicting TF targets using third party published data sets has been attempted in the past. Honkela et al. (2010) [13] provided a model-based method for TF target identification. It uses a differential equation model of transcriptional regulation to fit each putative target gene and rank the targets based on model likelihood. Cui et al. (2014) [14] generated an improved approach to predict TF targets based on the support vector machine. It uses a reverse-complementary distance-sensitive n-gram profile algorithm to convert each upstream sub-sequence into a high-dimensional vector data point. Subsequently, it converts prediction tasks into classification problems using an SVM classifier. Kim et al. (2006) [15] implemented a SVM classifier for miRNA target gene prediction. It uses three categories of features, structural features, thermodynamic features and position-based features, to represent the miRNA information. These are related works but none of these published works tackles the problem of pathway extension through TF target prediction in the way cTAP aims to solve.

The idea of using Z score of gene expression intensity values in addition to the log2FCs of test vs. control samples was motivated by closely observing the way scientists evaluate pathways’ enrichment scores. Although log2FCs is an important measure, biologists tend to assess the absolute amount of transcripts detected for a given gene in the test sample in judging the cell’s functionality in the system. Basically, the absolute amount of transcript present in the sample should also be factored in the decision making beyond the log2FCs. This issue becomes even more critical in single cell analysis, since the cell sub-type identification of the clusters is usually done by measuring abundance levels of known cell type marker genes. A remaining question however is, when should we call the amount of a gene’s transcript sufficient enough, even if its absolute amount is lower than that of the control case? Likewise, when should we call the gene’s transcript amount not sufficient enough even when it is higher than that of the control case? Relying only on p-values derived from log2FCs omits the possibility of exploring additional scenarios of gene regulation which are subtle but important.

## Results

**Fig. 1:**
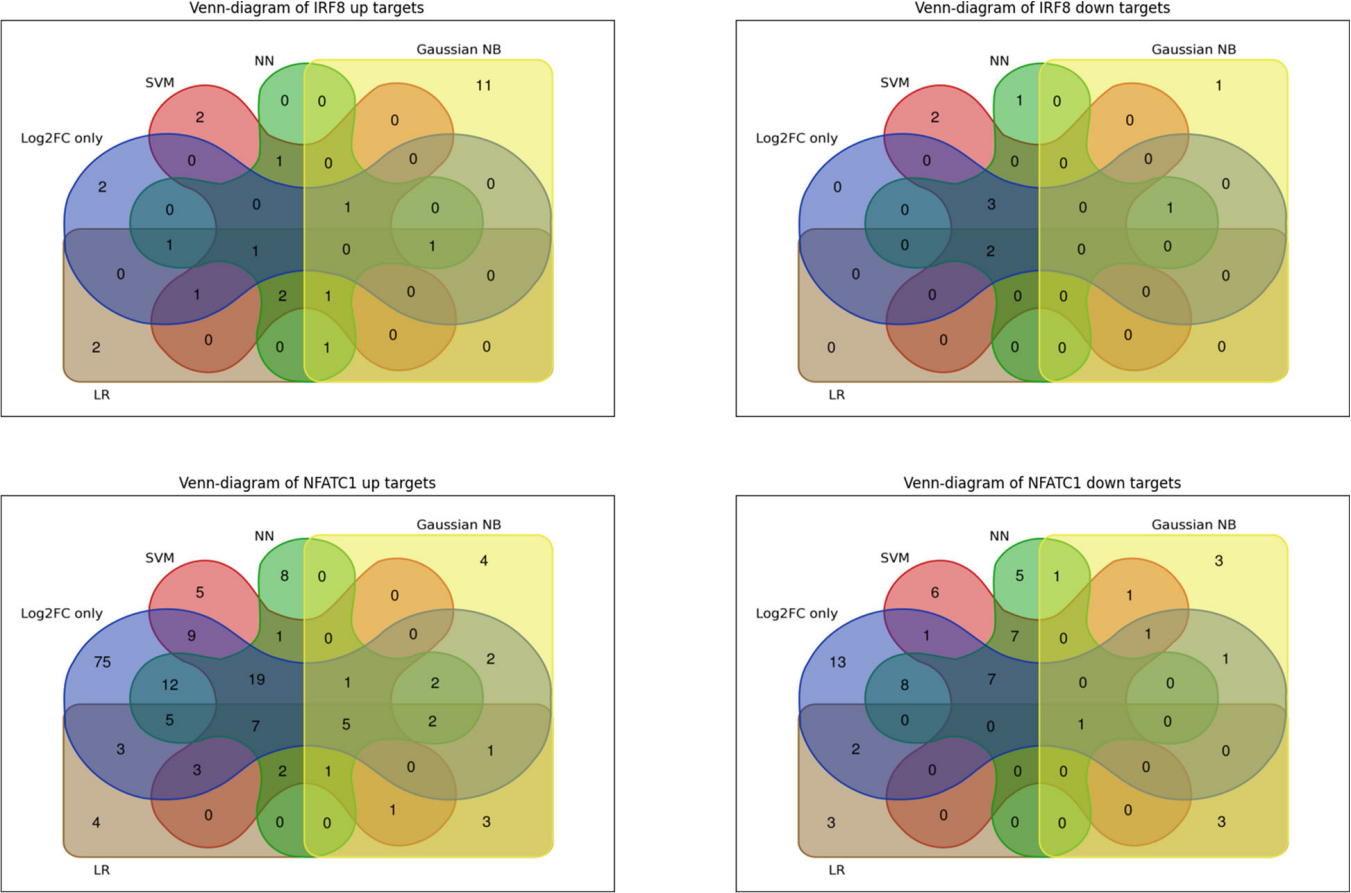
The Venn-diagrams of all targets, up targets and down for IRF8 and NFATc1

The results for predicting IRF8 and NFATc1 target genes are shown in Tables 1 and 2, respectively. The selected genes were obtained by applying all four ML models including SVM, NN, Gaussian NB and LR and were compared to the standard algorithmic method, Log2FC only. The Venn-diagrams shown in Fig. 1 compare the extent of overlap between these different methods for two separate cases; one for upregulated target genes (Up Targets) and the other for down regulated target genes (Down Targets). We performed a literature survey for the identified target genes for IRF8 and NFATc1. The goal of the survey was to examine if any of the identified target genes are already known for “osteoclast differentiation” in order to develop confidence in what cTAP produces. Overall, the result of predicting IRF8 targets by all three models follows our expectation. Each method predicted unique gene regulatory patterns, but most of them are overlapping as shown in Fig. 1. One noticeable distinction is the use of Log2FC only method to predict NFATc1’s target genes. Log2FC only, produced a much higher number of Up and Down Target genes compared to the other two ML based models. However, the genes selected by log2FC only did not show up in our literature survey, thus it remains unclear whether they have a role in bone remodeling or osteoclasts differentiation. For example, IL10RA, PRKD2, RBM43, RPS6KA3, TPD52, and ACADM are reported exclusively by the Log2FC only method, but no bone remodeling related studies have been reported for these genes. This finding possibly suggests that Log2FC only gene selection is prone to generating “false positive” cases. Below we summarize what we believe are “true positive” cases predicted by the ML models.

**Table 1:** IRF8 target gene regulation prediction based on the regulation prediction using SVM, NN and log2FC only

Model	Up regulation	Down regulation
**Log2FC only**	AIF1, CD164, MARCKS, MEF2C, RNASE4, TLR6, LSP1	NDUFS7, RAB3IP, NUDT13, MCRS1, COX15, ATP5L
**SVM**	AIF1, BID, CASP1, CTPS2, H2-M3, IRF5, LSP1, SLC15A3, TLR6	NDUFS7, PARP8, NUDT13, MCRS1, COX15, ATP5L, ALG9
**NN**	AIF1, BID, CTPS2, MARCKS, RNASE4, H2-M3, SLC15A3, NOTCH1, LSP1	NDUFS7, RAB3IP, NUDT13, MCRS1, TAP2, COX15, ATP5L
**Gaussian NB**	ATP6V0A1, CCL6, CCRL2, FGD2, LSP1, LY86, NOTCH1, P2RY12, PLSCR3, RNASE4, SLA, SLC15A3, STAT2, TNFRSF13B, TRIM21	GTF3C5, RAB3IP
**LR**	AIF1, CTPS2, H2-M3, MARCKS, NOTCH1, RNASE4, S100A13, SLC15A3, TLR6, TNFRSF1B	ATP5L, NDUFS7

**Table 2:** NFATc1 target gene regulation prediction based on the regulation prediction using SVM, NN and log2FC only

Model	Up regulation	Down regulation
**Log2FC only**	ABCB4, ACADM, ACADS, ACADVL, ACAT1, ACBD6, ACO2, AK2, ALDH4A1, AP1G2, APBA3, ATP5E, ATP5G1, ATP5G2, ATP5G3, ATP5J, ATP5L, ATP6V0B, ATP6V1C1, ATP6V1D, ATP6V1H, BCAT2, BCL2L13, BSG, C1QBP, CIAPIN1, COMTD1, COQ6, COX15, COX5B, COX7A2, COX7A2L, CS, CTTN, CYC1, CYCS, DLAT, DLST, ECSIT, ETFA, ETFDH, EXT1, FAHD1, FASTK, FDXR, GNB1L, GPX4, GSS, GTF2H3, HAGH, HINT3, IDH3A, IDH3B, IK, IMMT, KIF13A, LETM1, LRPPRC, MANBAL, MCAT, MCRS1, MDH1, MDH2, MFN2, MRPL1, MRPL14, MRPL34, MRPL36, MRPL38, MRPL46, MRPL48, MRPL51, MRPL9, MRPS25, MRPS34, MRPS35, MTX2, NDUFA10, NDUFA4, NDUFA5, NDUFA6, NDUFAB1, NDUFAF1, NDUFB10, NDUFB3, NDUFB5, NDUFB6, NDUFB7, NDUFC1, NDUFS3, NDUFS7, NDUFS8, NHEJ1, NSMAF, NUDT8, OGDH, OPA1, OXNAD1, PABPC4, PGAM5, PGLS, PIGO, PIP5K1B, PITPNM1, PPA2, PPARGC1B, PTGES2, PTPN12, PTPN9, RABGEF1, RELB, REPIN1, RREB1, SDHD, SLC25A11, SLC25A19, SLC25A3, SLC25A39, SLC25A5, SLC30A6, SLC39A13, SOD2, ST5, STARD7, TANK, TARBP2, TAX1BP3, TBC1D10B, TBRG4, TCIRG1, TERF2IP, TFRC, TIMM17A, TIMM44, TMEM60, TNFAIP3, TTC19, TUFM, UBE2G1, UBLCP1, UQCRC1, UQCRC2, UQCRH, USP4, VDAC1, VTI1B	CD164, CD48, CHST12, CNR2, CORO1A, EBI3, EPS15, FLI1, GCA, GNG2, IER3, IL10RA, IRF8, LAMP1, LSP1, MARCKS, NEDD9, NUCB2, P2RY6, PKIB, POU2F2, PRKD2, RASSF5, RB1, RBM43, RNASE4, RPS6KA3, SSBP2, TLE3, TLR6, TNFSF9, TPD52, WSB1, ZFP90
**SVM**	ABCB4, ACAD10, ACADS, AP1G2, ATP5L, CIAPIN1, CNIH4, COX15, COX7A2, CYC1, DLAT, DNAJA3, DUS3L, EXT1, FAHD1, FDXR, FEM1A, HAGH, HINT3, IDH3A, IMMT, LRPPRC, MANBAL, MCAT, MCRS1, MRPL36, MRPL38, MRPL9, MRPS34, MRPS35, NDUFB10, NDUFB5, NDUFB6, NDUFS3, NDUFS7, NSMAF, PABPC4, PEX16, PGLS, PIGQ, PIP5K1B, PPARGC1B, PRDX3, PTGES2, PTPN12, SEMA7A, SLC25A19, SLC39A13, SSNA1, ST5, TIMM44, TTC19, TUFM, USP4	ANXA6, ATF3, CCL3, CD48, CDC42EP3, CHST12, CPEB2, DCK, EBI3, FLI1, GNG2, IRF5, IRF8, LSP1, LXN, MAP4K2, PIK3CG, SLC15A3, SLC9A3R1, SP100, TEX2, TLR6, TNFSF9, ZFP90
**NN**	ABCB4, ACAD10, ACADS, AK2, AP1G2, ATP5G3, ATP5L, BCAT2, CIAPIN1, COG8, COQ6, COX15, COX17, CYC1, DLAT, DNAJA3, DUS3L, ECSIT, ETFDH, EXT1, FASTK, FDXR, GSS, HAGH, HINT2, HINT3, IDH3A, LRPPRC, MANBAL, MBTPS1, MCRS1, MRPL12, MRPL38, MRPL9, MRPS25, MRPS34, MRPS35, NDUFB10, NDUFC1, NDUFS3, NDUFS7, NSMAF, NUDT8, OXNAD1, PABPC4, PIP5K1B, PITPNM1, PPARGC1B, PRDX3, PTDSS2, PTGES2, RABGEF1, RELB, SLC39A13, STARD7, TANK, TAX1BP3, TBRG4, TCF12, TTC19, TUFM, UBE2G1, UBLCP1, UMPS, USP4	ADAM15, CCL3, CD48, CHST12, CPEB2, EBI3, FLI1, GNG2, IL10RA, IRF8, LSP1, LXN, MAP4K2, MARCKS, MS4A7, NAB2, NOTCH1, NUCB2, P2RY6, POU2F2, RB1, RNASE4, RPS6KA3, SLC15A3, SLC9A3R1, SNAP29, TEX2, TMBIM1, TNFSF9
**Gaussian NB**	ACO2, ACSL1, DLAT, DNAJA3, FDXR, GSS, MCRS1, NFKB2, NFKBIE, NUDT8, OTUD7B, OXNAD1, PPARGC1B, PTGES2, SARS2, SDC1, SEMA7A, SLC25A39, SLC39A13, TARBP2, TBRG4, TRAF1	CCR5, CDC42EP3, CNR2, ECE1, GSTK1, ICAM2, NAB2, PIAS3, SORL1, TLR6, TNFSF9
**LR**	ABCB4, ACAD10, AK2, AP1G2, ATOX1, ATP5G2, BCAT2, DNAJA3, DUS3L, EXT1, FAHD1, FASTK, FDXR, IDH3A, IVNS1ABP, MCRS1, MFN2, NDUFS3, NFKBIE, NSMAF, OTUD7B, OXNAD1, PABPC4, PPARGC1B, PTGES2, PTPN12, REPIN1, SDC1, SEMA7A, SERPINB8, SLC39A13, ST5, STARD7, TANK, TARBP2, TBRG4, XRCC5	GSTK1, HSPA2, ICAM2, NFKBIZ, PARVG, RASSF5, RBM43, SORL1, TNFSF9

First, we note that the AIF-1 gene (Table 1) has been called by all three methods as an Up target of IRF8 and the involvement of AIF-1 in osteoclast cells has been well established [16]. Additionally, Kimural et al. (2007) reported that AIF-1 induces the proliferation of cultured synovial cells, and it plays an important role in chronic immune inflammatory processes involving macrophages, the cell type in which IRF8 is known as a master regulator.

Similarly, the ABCB4 gene (Table 2) has been identified as an Up target of NFATc1 by all three models. The role of ABCB4 during osteoclastogenesis has been previously reported [17]. Irie et al. (2017) showed that ABCB4 expression was markedly increased during osteoclastogenesis. In contrast, its knockdown in pre-osteoclasts led to a reduction in osteoclast fusion [17].

Even for the genes that were uniquely called by only one of the two machine learning methods, their function has been implicated in bone remodeling as summarized below. While our literature survey identifies gene associations with bone modeling, it does not determine whether specific genes are directly regulated by either IRF8 or NFATc1. However, since our analysis utilizes ENCODE ChIP-Seq data, we hypothesize that the identified genes could be direct targets of these two transcription factors. Nevertheless, our analysis is predictive and hypothesis generating; wet-lab experiments will be necessary to provide more definitive evidence that these regulatory mechanisms actually exist.

*BID* - The involvement of BID in regulating osteoclast formation has been previously reported [18]. RelA, a subunit of NF- *κ*B, has been shown to block the RANKL-induced JNK-BID apoptotic pathway and, by doing so, promotes OC differentiation. Our machine learning system placed BID in the Up target list of IRF8, suggesting that the expression of BID may not increase if IRF8 is suppressed. BID is known for its role in regulating apoptosis [19]. When this fact is combined with IRF8, itself playing a role in inhibiting NFATc1, the additional regulatory relationship between IRF8 and BID likely suggests that IRF8 possesses alternative mechanisms to negatively regulate osteoclastogenesis.

*NOTCH1* - In the skeleton, Notch signaling can regulate the differentiation and function of both osteoblasts and osteoclasts. NOTCH1 can negatively regulate osteoclast formation indirectly by promoting the expression of osteoprotegrin in osteoblasts [20]. However, NOTCH1 also functions in osteoclast precursors to repress osteoclast formation [21]. Our method identifies NOTCH1 in the Up target list of IRF8 (Table 1), suggesting its suppressive role in osteoclast differentiation. Interestingly, our method also predicted NOTCH1 as a Down Target of NFATc1. Our findings are consistent with the well-known opposite roles that IRF8 and NFATc1 have in osteoclast differentiation but also implicate the existence of downstream mechanisms where NOTCH1 expression is tightly regulated positively and negatively, by IRF8 and NFATc1, respectively.

*IRF5* - Interferon regulatory factor 5 (IRF5) has an important role in the differentiation of myeloid derivatives from mouse bone marrow [22]. Our SVM model predicted that IRF5 may function as a repressor of osteoclast differentiation through the activation of IRF8. Consistent with this prediction, Yang et al.(2019) [23] reported that silencing IRF5 increased osteoclast differentiation. However, in this study, NFATc1 expression leads to suppression of IRF5 expression.

*CASP1* – Rocha et al. (2020) [24] reported that CASP1 and NLRP3 deficiency increases the activity of RANKL-derived osteoclasts. Our SVM model prediction placing CASP1 in the Down list of IRF8 suggests that CASP1 is down-regulated through IRF8 along with IRF8’s own down regulation when osteoclast differentiation increases. The placement of CASP1 in the Up target of IRF8 is consistent with what has been reported by Rocha et al.

*NAB2* - In [25], Kim et al. (2012) stated that EGR2 over expression can inhibit RANKL-induced osteoclast differentiation and also NAB2 binding to EGR2 can inhibit its actions to restore osteoclast differentiation. In our NN model prediction, NAB2 is placed as a Down Target of NFATc1. This discovery suggests an interesting negative feedback, albeit putative, possibly explaining maintenance of homeostasis in osteoclast differentiation. During osteoclasts differentiation, NFATc1 is known highly upregulated and that would suppress NAB2’s expression according to our model prediction. Since Kim et al. suggested that NAB2 inhibits EGR2’s suppressive role in upstream of NFATc1, a negative feedback loop is established. That is, during the suppressive state of osteoclast differentiation, NAB2 can over express to repress EGR2 which will subsequently allow RANKL to induce osteoclast differentiation through NFATc1 over expression. When NFATc1 becomes over expressing, it suppresses NAB2 to reverse its binding to EGR2, thus allowing EGR3 to inhibit osteoclast differentiation.

*TCF12* - In [26], Yi et al. (2017) reported that overexpression of TCF12 in mesenchymal stem cell suppresses the osteoblast differentiation. Our NN model places TCF12 as an Up target of NFATc1 which makes this prediction consistent with the TCF12’s role in osteoclast differentiation reported in [26]. In addition, Putt et al. (2009) [27] stated that TCF12 is a co-regulator of NFAT family and MEF2 family in heart failure disease, possibly suggesting a common regulatory relationship between TCF12 and NFATc1 across multiple tissue types.

In summary, we reiterate the two facts. First, many discovered targets are already known in the osteoclast literature although some do not have direct reference to bone remodeling, suggesting potential validity of the results produced by the learned models as well as discovery of possible novel targets. Second, none of the articles is explicit in stating the role of IRF8 and NFATc1 in their studied systems, suggesting the potential value and novelty of newly discovered “direct” regulatory relationships between IRF8, NFATc1 and their identified targets in osteoclast differentiation.

## Methods

**Fig. 2:**
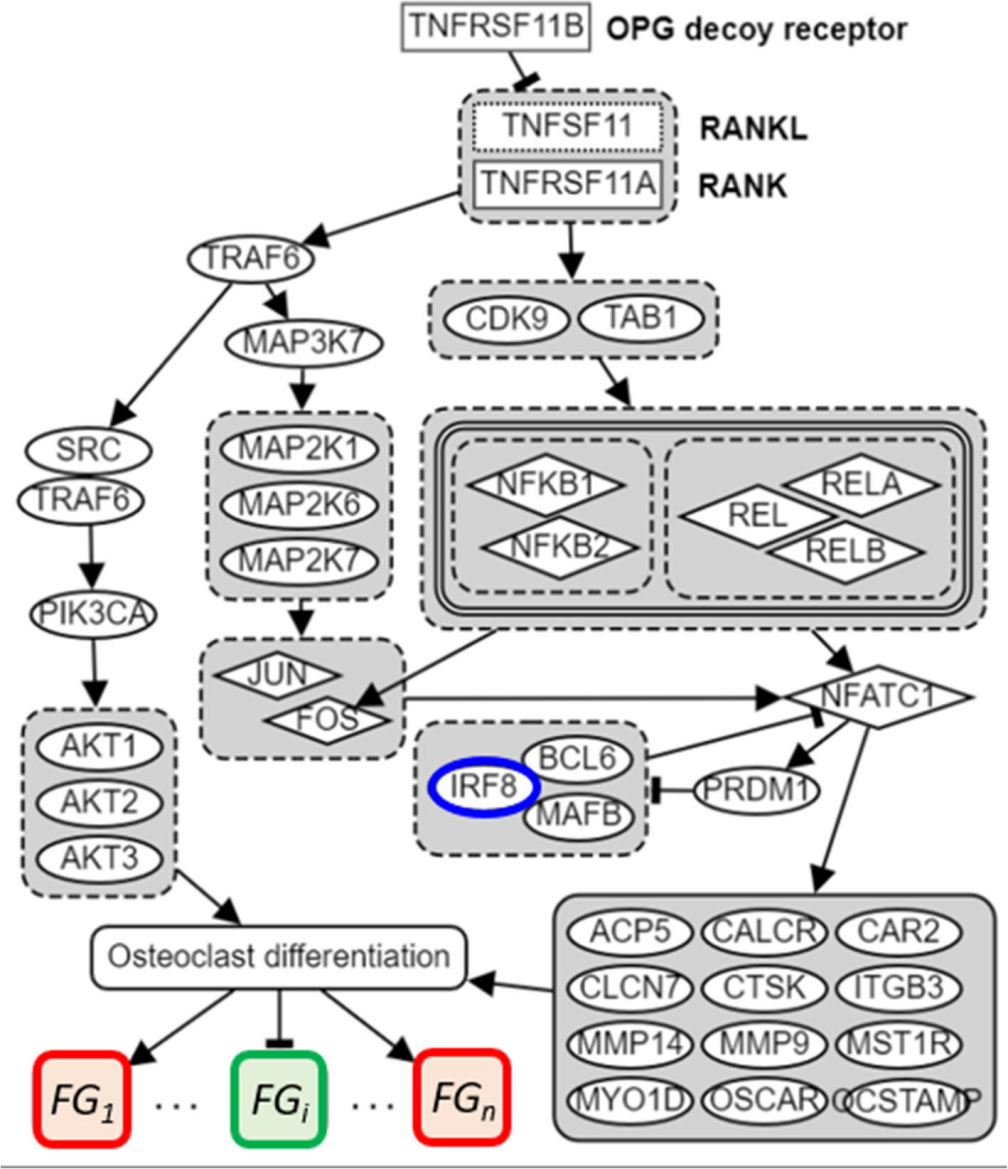
The overall process of cTAP

The overall framework of cTAP is given in Fig. 2. The figure outlines how multiple analysis steps are integrated to make the final TF target prediction. The framework is made up of three major components, assembling “Comparison-Pairs” (CPs) designed to generate a cohort of gene expression data sets specific for osteoclast differentiation, introducing “Functional Groups” into the regulatory pathway constructed to encode gene and their relationships known for osteoclastogenesis, and training and using the learning model for TF target prediction.

### Generating comparison pairs

**Fig. 3:**
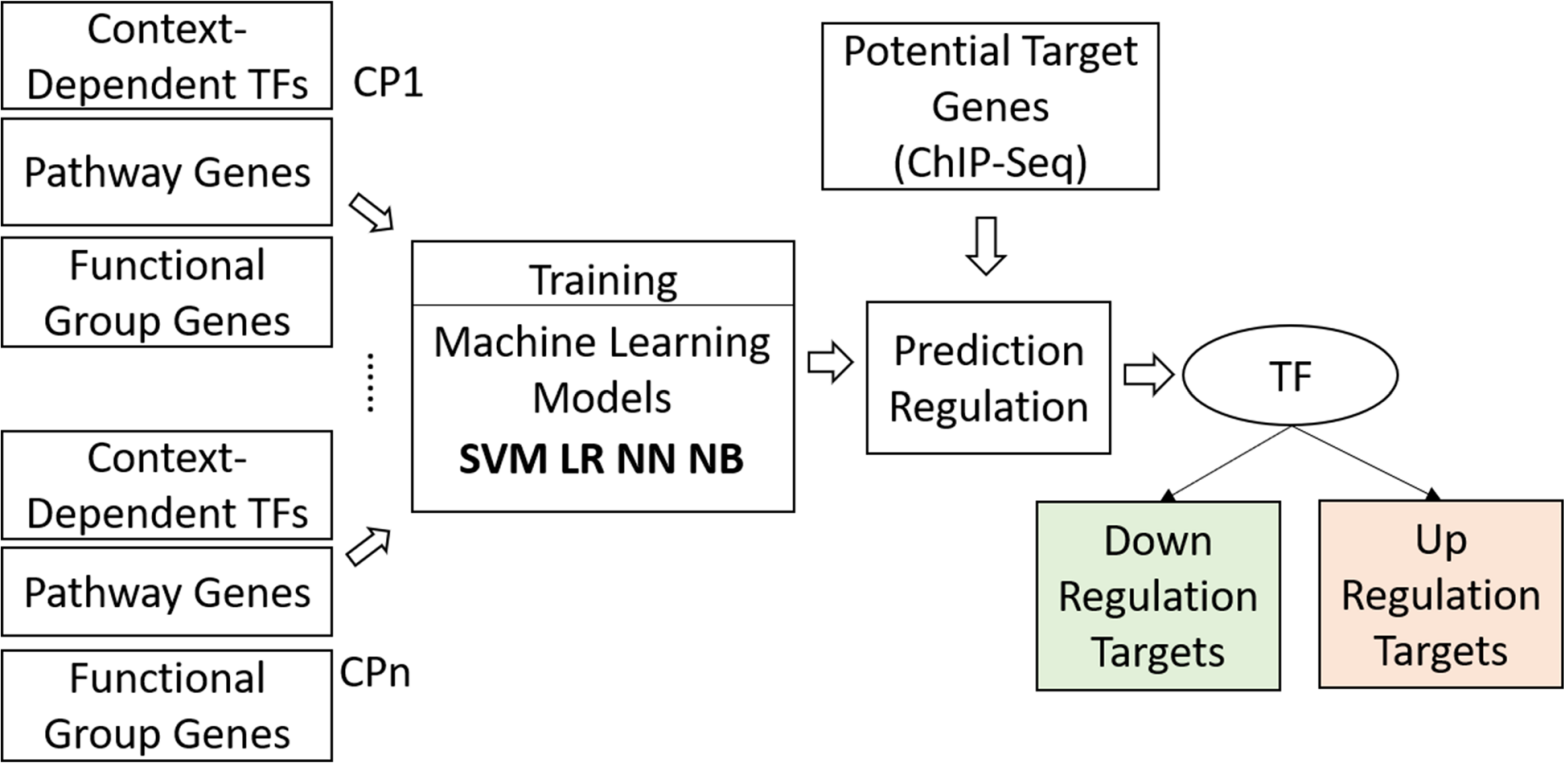
CPs examples illustrating the issues and complexity involved in determine which pair of gene expression populations should be chosen for the analysis. (a) “No RANKL” vs. “RANKL” for OCU and “RANKL” vs. “RANKL with LEA” for OCD. (b) “osteoclast progenitors” vs. “mature-osteoclast progenitors” for OCU

The first step of cTAP is identifying and creating “Comparison Pairs” (CPs) from GEO published data sets that are relevant to the concerned biological context, i.e., osteoclast differentiation in our case. Building CPs requires biological knowledge to identify which pairs of samples in the given study population should be compared with each other. One important requirement is to identify two sets of CPs whose functional regulations convey the opposite direction, one for up regulation and one for down regulation, within the concern context. For example, in our case, one should be osteoclast differentiation up regulation (OCU) and the other should be osteoclast differentiation down regulated (OCD). Figure 3 displays this concept by using two example data sets, GSE 142866 in Fig. 3a and GSE111237 in Fig. 3b. GSE142866 is a simple study that examines the impact of LEA on bone remodeling. From this, one can identify two CPs, one comparing the samples of “No RANKL” with “RANKL” for OCU and one comparing “RANKL” with the combination of “RANKL and LEA” for OCD. GSE111237 is a bit more complex study that involves six populations, each having 3 technical replicates, making total 18 samples. In this case, total 8 CPs are possible but not all comparisons should be used. In fact, in this GEO data set, we considered that only one CP is relevant to our cohort study, i.e., comparing “ostecoclast progenitors” with “mature osteoclast progenitors” as OCU. Biologists generally do not compare cases that involve more than one variable due to difficulties in interpreting the outcome. For example, comparing Era-pre-osteoclast progenitors and mature-osteoclast progenitors involves both estrogens and cell type and thus should be avoided. When comparing gene expression values between test and control populations, its goal is to obtain information regarding how the involved variable discerning test vs. control could impact gene regulation. In case more than one variables are involved, parsing why and how the changes in gene regulation occur becomes too complicated.

The format of CP is a two-column matrix which contains all gene symbols and their corresponding log2 ratios. In general, comparing gene expression values between test and control populations provides information on gene regulation. A gene with a higher expression value in the test than the value in control sample is supposed to mean up-regulation with its log2 ratio being positive. In contrast, a gene with a lower expression value in the test than the values in control sample is supposed to mean down-regulation with its log2 ratio being negative. The following steps describe the detail of generating CP from the series matrix file of GE data that is downloaded from GEO:

- Duplicate samples in the series matrix file are grouped into populations. A CP is formed by pairing up a test population and a control population. Its context is determined based on the experiment variables and their effect.
- Log2 ratio (*R*_*i*_) for each gene *i* is calculated using the following Eq. 1. *E*_*t*_ and *E*_*c*_ stand for the populational average expression value of the gene in the test and control population, respectively. Laplacian correction is applied to reduce the effect of small expression values. *E*_*t*_ and *E*_*c*_ are increased by 0.01 while taking the ratio.
  1 $$ \begin{aligned} R_{i} = {log}_{2}\left(\frac{E_{t}+0.01}{E_{c}+0.01}\right) \end{aligned}  $$

### Modeling functional group

**Fig. 4:**
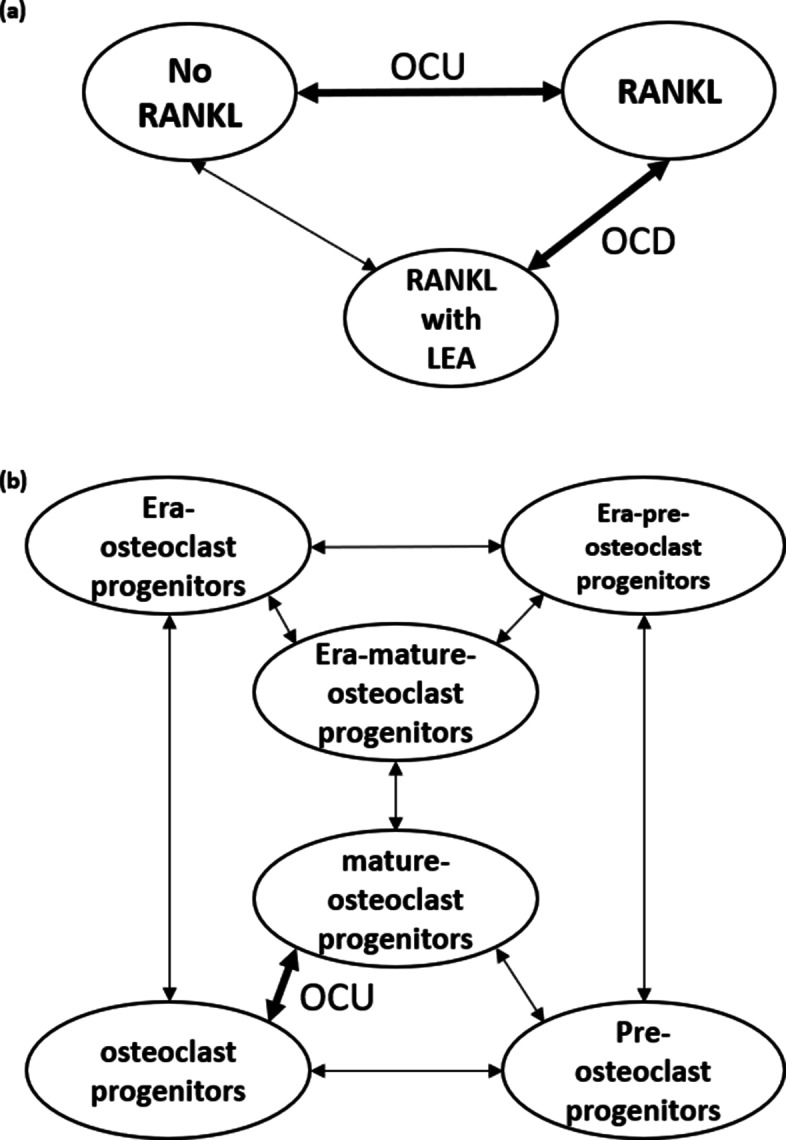
Osteoclast differentiation pathway diagram including IRF8, NFATc1 and functional groups of marker genes

Introducing functional groups into the pathway is to overcome the limitation of the existing pathway analysis systems. Pathway analysis has been popular to gain insight into the underlying biology of differentially expressing genes and proteins as it reduces the complexity of the analysis. Khatri et al. (2012) [28] summarized that there have been three generations of pathway analysis methods: Over-Representation Analysis (ORA), Functional Class Scoring (FCS) and Pathway Topology (PT) - Based analysis. Examples of each system are, respectively, GOstat [29], GSEA [30] and SPIA [31]. PT-Based systems outperform others as it provides the visual understanding of gene regulation patterns when log2FC are overlaid over the pathway diagrams. However, even in the well-known curated pathway system like KEGG [32], only a limited number of target genes in downstream of a transcription factor (TF) are included. For example, in KEGG Osteoclast differentiation pathway (Entry ID: hsa0480), only four genes, CTSK, TRAP, CTR and *β*3 integrin, are included in the downstream of NFATc1, the key TF known regulating osteoclast differentiation. Thus if we are interested in inferring the targets of IRF8 within the osteoclast differentiation, using this pathway is impractical. Our approach is that functional groups specific to osteoclast differentiation is included in modeling a pathway as we illustrate such plan in Fig. 4. In this figure, osteoclast differentiation is characterized by having n functional groups (FGs), each of which including a set of genes that are known to either collectively activated or collectively suppressed in the context of osteoclast differentiation. In our study we have included about ∼134 such curated genes in 14 functional groups and we use them for the discovery of targets of IRF8 and NFATc1 in the osteoclast differentiation context.

Each functional group (FG) is a collection of marker genes that should collectively work towards a biological process in one functional direction, i.e., either all up-regulated or all down-regulated. What genes should be included in each FG is determined through a curation effort and discussing how it is done is beyond the scope of this paper. Ideally, all genes included in each FG should be all up or down-regulated for a given context. However, in a biological system such agreement occurs rarely, and the degree of regulation agreement over a certain threshold (e.g., over 80%) should be considered sufficient.

In order to quantify the degree of agreement/disagreement of activation and inhibition of a FG, we introduce the notion, called, FG score (FGS). A negative score means FG is inhibited, and a positive score means FG is activated. The more genes within a FG is up-regulated, the more activated the FG is. The more genes within a FG is down-regulated, the more inhibited the FG is. Equation 2 is used to calculate the *j*^*th*^ FGS. Let *R*_*i*_ be the log2FC for genes in FG and *n* be the number of genes in that FG. The total functional group score (TFGS) is defined as the number of FGs that follow the expected functional state associated with all CPs. For instance, if there are two CPs, one of them has 5 FGs and the other 6 FGs following the expected behavior, then TFGS of these two CPs is 11

2 $$ {FGS}_{j} = \left\{ \begin{aligned} \left(\frac{ \sum_{n}^{i} R_{i} }{n} \right)^{2} &&&&&&&& if &&& \left(\frac{ \sum_{n}^{i} R_{i} }{n} \right) \geq 0 \\ - \left(\frac{ \sum_{n}^{i} R_{i} }{n} \right)^{2} &&&&&&&& if &&& \left(\frac{ \sum_{n}^{i} R_{i} }{n} \right) < 0 \end{aligned} \right.  $$

### Training and prediction

**Fig. 5:**
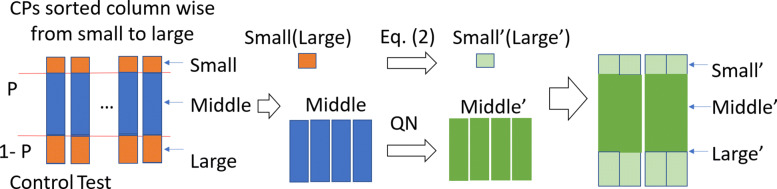
The overall process of trimmed quantile normalization
- Ignore the order of gene symbols and cut off minimum (small value genes) and maximum (large value genes) with *P* percent from every column. Perform quantile normalization on the combined remaining parts (the middle part) of the columns.
- Change the small and large parts of each column using the Eq. 3. *Vi*′ represents the revised value for gene *i* in the small or large value genes. *Z*_*m*_ is the column-wised Z score in the matched middle part. *N* is the number of genes whose Z scores are positive. *n*^′^ represents the number of genes from *i* to the gene whose Z-score is 0 in the middle part. Here *σ* and *μ* are the standard deviation and average of the middle part, respectively.
- Combine the revised top and bottom parts with the normalized middle part. Then add the gene order for each column to the columns.
- Reorder each column to make same gene appear in same row, and by doing so generate the normalized matrix (*M*^′^).

The first step of this part is to combine GEO data sets from different laboratories into a cohort of CP arranged data sets. One difficulty in this data munging step is how to handle the biases originating from different platforms used by individual laboratories. Quantile normalization is a popular way to normalize data, but it tends to produce skewed results if some samples have extreme values. Extreme values affect the average value and makes the normalized data biased. In cTAP, we use a trimmed quantile normalization (TQN) to overcome this issue. It takes an unnormalized big matrix (*M*), which is generated based on control and test populations averaged expression values from all CPs as an input where every CP having two column values, one for control population and one for test population. Value 0 is used to impute the missing genes in the population. Since the minimum and the maximum value for each column in *M* will be removed temporarily before quantile normalization, imputing the missing value with 0 does not impact the performance of TQN. The overall process of TQN is shown in Fig. 5. The detailed steps are described below:

- Order gene expression value in ascending order for every column (sample) and save the order of different gene symbols for each column.

3 $$ \begin{aligned} V_{i}^{\prime}= \left\{ \begin{aligned} \left| \frac{ min(Z_{m})}{N} \right|*n^{\prime}*\sigma + \mu &&&&& small && values \\ \left| \frac{ max(Z_{m})}{N} \right|*n^{\prime}*\sigma + \mu &&&&& large && values \end{aligned} \right. \end{aligned}  $$

To quantify the notions, Gene-Present Sufficiently (GP) and Gene-Absent Insufficiently (GA), we use the cohort’s gene-wise Z-score as the tool. Total 4 types of Z-scores for gene *i* are computed based on *M*^′^. They are row-wise (gene across samples) Z-scores and column-wise (sample across genes) Z-scores for both test and control populations, denoted by (*Z*_*Ric*_, *Z*_*Rit*_, *Z*_*Cic*_, *Z*_*Cit*_). Equation 4 is used to calculate row-wise Z scores, *μ*_*R*_ and *σ*_*R*_, respectively, denoting the average and the stand deviation for the row values. Likewise, Eq. 5 is used to calculate the column-wise Z scores, *μ*_*C*_ and *σ*_*C*_, respectively, denoting the average and the stand deviation for the column values.

4 $$ \begin{aligned} Z_{Ri} & = \frac{V_{i} - \mu_{R}}{\sigma_{R}} \end{aligned}  $$

5 $$ \begin{aligned} Z_{Ci} & = \frac{V_{i} - \mu_{C}}{\sigma_{C}} \end{aligned}  $$

The next step is using key genes in the pathway diagram (from FG and related TFs) and *M*^′^ to build a learning model which is capable to determine if a potential target of the TF can be classified into either GA or GP. Here, GA means that gene transcript abundance is “insufficient” meaning its transcript amount is too low to exert its intended function in the system of the test sample. A gene’s GA status in the test sample may override its log2FCs being positive. GP means that gene transcript abundance may be “sufficient” meaning its transcript amount is high enough to exert its intended function in the system of the test sample. A gene’s GP status in the test sample may override its log2FCs being negative.

The basic idea behind cTAP is similar to the existing works that mentioned before because it relies on the learned model to call if a gene is highly likely a target or not. But our work is unique in that it can call if a gene is either an Up target or a Down Target. Such decision is possible in our approach as we use the input data defined for opposite regulatory contexts, i.e. OCU and OCD. In Table 3, an example case in which Caspase-1 (CAPS1) is down-regulated in most of OCU CPs and up-regulated in most OCD CPs. Another uniqueness in our approach is that it uses the learned model’s classification of GP and GA for each potential target gene. We introduce a parameter, called the error tolerance rate (*T*), to control the error in determining the target, i.e., how much of inconsistency should be allowed in cTAP’s decision making. In CASP1 case in Table 3, *T* was set to 15%. Even if the regulation pattern of CASP1 does not agree with that of IRF8 in two of the CPs (CP ID: 5 and 16), it is still considered as down-regulation in OCU CPs.

**Table 3:** CASP1 downregulated in most of OCU CPs and upregulated in most of OCD

ID	Context	CASP1 log2FC
1	OCU	-0.128
2	OCU	-0.400
3	OCD	0.495
4	OCU	-0.350
5	OCU	0.247
6	OCU	-0.861
7	OCU	-1.314
8	OCD	0.144
9	OCD	0.387
10	OCD	0.748
11	OCD	1.001
12	OCD	1.251
13	OCD	0.596
14	OCU	-1.034
15	OCD	0.494
16	OCU	0.069

The training data set is formed by context-related TFs and genes in FGs. Log2FCs and 4 types of Z scores for each gene make up five features for each training sample. In details, the first feature is the gene log2 folder changes computed using Eq. 1. For the second, third, forrth and fifth features of a gene are column-wised Z score of the control expression value, column-wised Z score of the test expression value, row-wised Z score of the control expression value, and row-wised Z score of the test expression value, respectively. How to compute these Z-scores has been discussed in the above. Gene’s expected regulation is used as the class label. The gene is labeled “1” if its expected regulation is up-regulation or “-1” if its expected regulation is down-regulation. One of the classifiers from SVM, Gaussian, LR and Neural Networks (NN) are selected to be trained. Once the model training is over, it is used to predict the regulation state of each potential target gene.

## Experiment and discussion

### Experiment materials

#### Choosing appropriate GEO data sets

In this study, we used gene expression studies that are publicly available at NCBI GEO (https://www.ncbi.nlm.nih.gov/) and that involve only osteoclast progenitors and mature osteoclast progenitors. Total data sets from 11 GEO studies were downloaded. The specific GSE IDs for the downloaded data sets are shown in Table 3. Each of these data sets is preprocessed to have its gene identifiers (called, ID_REF) mapped to official gene symbols using the accompanying annotation file available at NCBI GEO for each data set, namely Platform file, for example, using GPL6885 “Illumina MouseRef-8 v2.0 expression beadchip” for the data set GSE111237. The downloaded and preprocessed data sets are then subject to “trimmed quantile normalization (TQN)” as discussed in “Methods” section (Fig. 5) to enable that data sets generated by independent labs can be cohesively compared. Next step is generating “Comparison Pairs (CPs)” by closely examining the study context so that which subgroups of samples should be bundled and compared for our intended two types of comparison semantics, i.e., osteoclast differentiation up regulation (OCU) and osteoclast differentiation down regulation (OCD). For example, in case of GSE142866 in Table 3, comparing No RNAKL group as Control Population with RANKL group as Test Population is to create OCU as RANKL is the known ligand whose binding to RANK initiates signaling for osteoclast differentiation. On the other hand, comparing RANKL treated group as Control Population and RANKL with LEA as Test population is to create OCD as LEA is known repressing RNANKLE-mediated osteoclast differentiation. Similarly, in case of GSE72846, comparing RANKL as Control Population with MMP9 KO as Test Population creates OCD as the matrix metalloproteinase 9 is the principal H3NT protease of osteoclastogenesis and its KO hampers the progression of osteoclastogenesis and thus OCD. The summary of all CPs with short descriptions suggesting how Control/Test populations are built and what regulatory phenotype that comparison should produce (i.e., OCU/OCD) is given in Table 4. Overall, total 16 CPs, 8 CPs for OCU and 8 CPs for OCD, were produced from the 11 studies including GSE111237 [33], GSE142866 [34], GSE149887 [35], GSE152986 [36], GSE17563 [1], GSE20850 [37], GSE30160, GSE37219 [38], GSE57468 [39], GSE76988 [40], GSE72846 [41] and GSE135479 [42]. As noticeable, some studies include both OCU and OCD cases and some only one type.

**Table 4:** Total 16 CPs related with osteogenesis generated from 11 GEO data sets

ID	GSE ID	Platform	Control population	Test population	Context
1	GSE111237	GPL6885	osteoclast progenitors	mature-osteoclast progenitors	OCU
2	GSE142866	GPL17021	No RANKL	RANKL	OCU
3	GSE142866	GPL17021	RANKL	RANKL with LEA	OCD
4	GSE149887	GPL21103	Mo (macrophages)	Oc (osteoclasts)	OCU
5	GSE17563	GPL339	bone marrow treated with hRANKL 0 hr	bone marrow treated with hRANKL 24h	OCU
6	GSE17563	GPL339	bone marrow treated with hRANKL 0 hr	bone marrow treated with hRANKL 72h	OCU
7	GSE20850	GPL1261	Macrophages	Osteoclasts	OCU
8	GSE30160	GPL1261	WT	RANK IVVY Knockin	OCD
9	GSE37219	GPL8321	WT	NFATc1-deficient OC	OCD
10	GSE57468	GPL6885	BMM RANKL 1day	BMM RANKL 0day	OCD
11	GSE57468	GPL6885	BMM RANKL 2day	BMM RANKL 0day	OCD
12	GSE57468	GPL6885	BMM RANKL 3day	BMM RANKL 0day	OCD
13	GSE76988	GPL13112	wild-type osteoclast M-CSF RANKL 24H	wild-type osteoclast M-CSF RANKL IL-3 24H	OCD
14	GSE76988	GPL13112	wild-type osteoclast precursor M-CSF 24H	wild-type osteoclast M-CSF RANKL 24H	OCU
15	GSE72846	GPL17021	Control	MMP9 KO	OCD
16	GSE135479	GPL21103	RANKL	FOXO3 RANKL	OCU

#### Generating functional groups

**Fig. 6:**
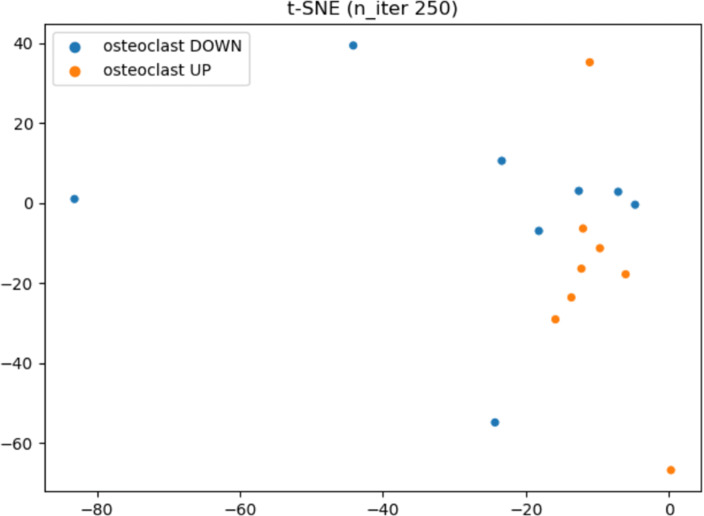
The 2-D plot produced by applying t-SNE to FGS of 16 CPs. Orange colored dots denote OCU CPs and blue colored dots OCD CPs

We assume that FGs are a priori known and have already been incorporated into the pathway diagram of the concerned biological context, as such has been illustrated in Fig. 4. We show an example of FGs that were built into osteoclast differentiation in Table 5. This table includes total 14 FGs made up of 134 genes: Secreted factors for external cells - Up (6 genes), Secreted factors for external cells - Down (13 genes), Coupling factors (7 genes), Integrin beta 3 (12 genes) Auto regulatory-up (6 genes), Auto regulatory-down (11 genes), Cytoskeleton control (7 genes), Acid and enzymes for matrix dissolution (15 genes), Cell differentiation (11 genes), Cell differentiation signaling factors - Up (9 genes), Cell differentiation signaling factors - Down (6 genes), Cell signaling (13 genes), MSC signature (12 genes) and Calciuren pathway (6 genes). Figure 6 displays a 2-D plot of 16 CPs using t-SNE to illustrate what will happen if we try to reduce the 14 FGSs to two features for each CP. This t-SNE plot shows that the separation between OCD and OCU cases is not obvious and suggests the limitation of using only gene value log2FCs to compute the gene’s functional state.

**Table 5:** Total 14 functional groups have different behaviors in different contexts

Functional group	Contained genes	OCU	OCD
1. Autoregulatory - up	CCL9, CCR1, CD109, CXCL10, SDC1, VEGFC	Activation	Inhibition
2. Cell Differentiation signaling factors - down	PLCB4, PLCB2, GIT1, DOCK5, TRAF1, TRAF6	Activation	Inhibition
3. Cell Differentiation	CLCN7, CAR2, CALCR, CSF1R, TREM2, TNFRSF11A, OSCAR, OCSTAMP, MST1R, ITGB3, DCSTAMP	Activation	Inhibition
4. Cytoskeleton Control	DCSTAMP, LAD1, MYO1B, OCSTAMP, SCIN, MYOD1, MARCKS	Activation	Inhibition
5. Integrin Beta3	CLCN7, OCSTAMP, CALCR, MST1R, CTSK, MMP14, ITGB3, MYO1D, ACP5, MMP9, CAR2, OSCAR	Activation	Inhibition
6. Secreted Factors for External Cells - up	INF2, SEMA4D, SGPL1, SPP1, CXCL10, CCL9	Activation	Inhibition
7. Coupling Factors	PGF,SPNS2,CD200,SGPL1,SEMA7A,LIF,CST7	Activation	Inhibition
8. ACID & Enzymes for Matrix Dissolution	VCAN, ATP6V0D2, CAR2, CLCN7, SLC9B6, CTSK, ACP5, PDE2A, MMP14, HTRA1, MMP9, ADAM10, ATP6V0B, ATP6V0C, ATP6V0C-PS2	Activation	Inhibition
9. Autoregulatory - down	C1QA, C1QB, C1QC, CCL2, CCL3, CCL4, CCL6, CCL7, CXCL14, IGFBP4, PF4	Inhibition	Activation
10. Calcinuren Pathway	CALM1, CAMK1, CAMK2A, CALM2, CALM3, PPP3CA	Inhibition	Activation
11. Cell Differentiation signaling factors - up	PPP2R3A, PPP3CA, CALM2, PPP2R3C, CAMK1, TNFAIP2, CAMK2A, CALM3, CALM1	Inhibition	Activation
12. Cell Signaling	SLIT1, SGPL1, INFB, IL10, CXCL5, IGF1, SPP1, SLIT3, C1QA, CCL8, CCL7, C1QC, C1QB	Inhibition	Activation
13. MSC Signature	ACTA2, ACTG2, BGN, CCND1, COL1A1, COL1A2, COL2A1, DKK3, FN1, SERPINH1, SPARC, TNC	Inhibition	Activation
14. Secreted Factors for External Cells - down	CCL6, CCL4, CCL3, CCL2, C1QC, C1QB, CCL7, CXCL14, CD200R1, CXCL16, IGF1, APOE, C1QA	Inhibition	Activation

#### Choosing key genes and incorporating ChIP-seq identified targets

Since IRF8 and NFATc1 play an essential role in bone metabolism as mentioned earlier, these two TFs are included in the training set along with the genes identified in FGs. The training is done using the class labels, OCU and OCD, as summarized in Table 5. In addition, total 213 ChIP-seq identified putative IRF8 target genes obtained from mouse GC B cell lines [23] and 6,812 ChIP-seq identified NFATc1 target genes from Harmonizome [43] have been fed into the learned models.

### Training and evaluation

For computational experiments, we used four well-established machine-learning models, SVM, LR, Gaussian NB and NN, to classify gene regulation patterns and compared their performances. All four models were implemented using scikit-learn [44] and in each case, grid search method [45] was applied to optimize the hyper parameters to deliver the highest AUC. For SVM, four kernel functions of different types, linear, nonlinear, polynomial, radial basis function (RBF) and sigmoid, were tested. The linear kernel function was selected for the comparative study because it performed the best while the sigmoid kernel function performed the worst. In case of Gaussian NB model which a variant of Naive Bayes model, the parameter only affects the calculation stability and thus default parameter setting in scikit-learn was used. In case of LR model which uses the trained logistic function to compute the probability of the default class, l1 penalty was used to get the highest AUC. For NN, a multi-layer perceptron classifier with two hidden layers was built with its first hidden layer having 5 nodes and the second one having 3 nodes. For its activation function relu function was used and adam optimization was applied for the training.

Each training set includes the five features for each of 134 genes in FGs and the two TFs, IRF8 and NFATc1. The training set has 2,176 (=136*16) samples from 16 different CPs. Among them, 70% of samples were used to train the models, and 30% were used to produce the ROC curves. To compare the performance of the learned models with that of the baseline case, the same number of genes not from FGs were randomly selected from CPs with the same label assigned with each FG. For instance, a random gene with its 5 features is fed to train the model without changing the label obtained from its membership belonging to its FG.

**Fig. 7:**
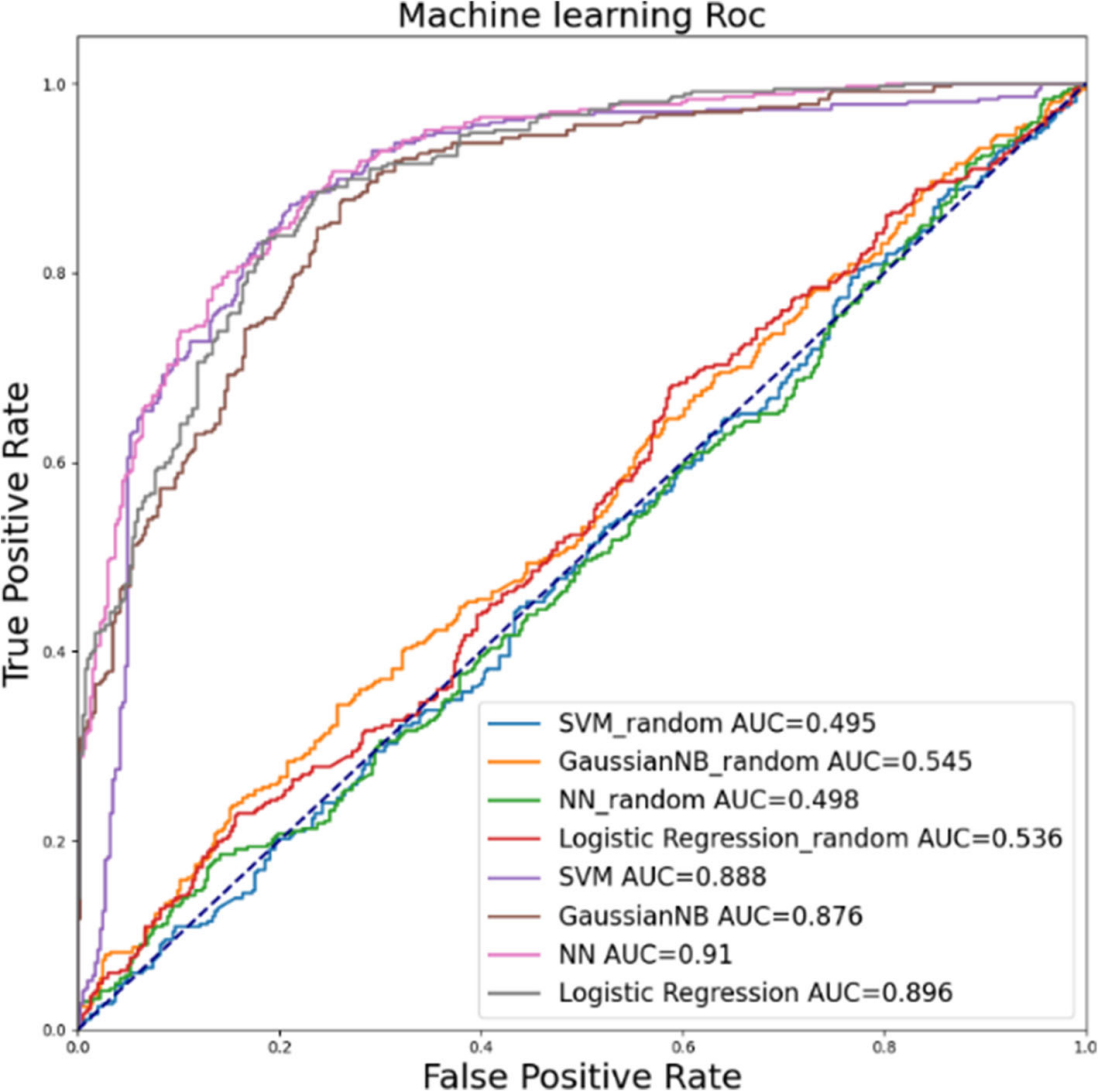
ROC curve of 4 different models’ prediction results using genes in FGs and random genes

ROC curves are shown in Fig. 7. All ROC curves using functional group genes have AUC area above 0.85 and the models using random genes have AUC area around 0.5 as anticipated. This evaluation clearly suggests that the learned models were able to make a reasonably accurate prediction for the target gene’s regulation state by using the input gene’s 5 features. Other model performance measurements including Accuracy (ACC), Matthews correlation coefficient (MCC), Sensitivity (SN) and Specificity (SP) are also shown in Table 6. Among the four models, SVM, LR and NN have a similar ACC and MCC while Gaussian NB has a relatively lower ACC and MCC. All models have similar specificity. Considering the lower value of the AUC and ACC, Gaussian NB seems inferior in its capability to contrast label-driven data difference compared to the other models

**Table 6:** Performance of 4 models

Models	ACC	MCC	Sn	Sp	TP	TN	FP	FN
SVM	0.79	0.58	0.82	0.76	303	283	87	65
Gaussian NB	0.69	0.41	0.48	0.89	177	332	38	191
NN	0.81	0.61	0.84	0.77	310	285	85	58
LR	0.79	0.59	0.78	0.81	288	300	70	80

**Fig. 8:**
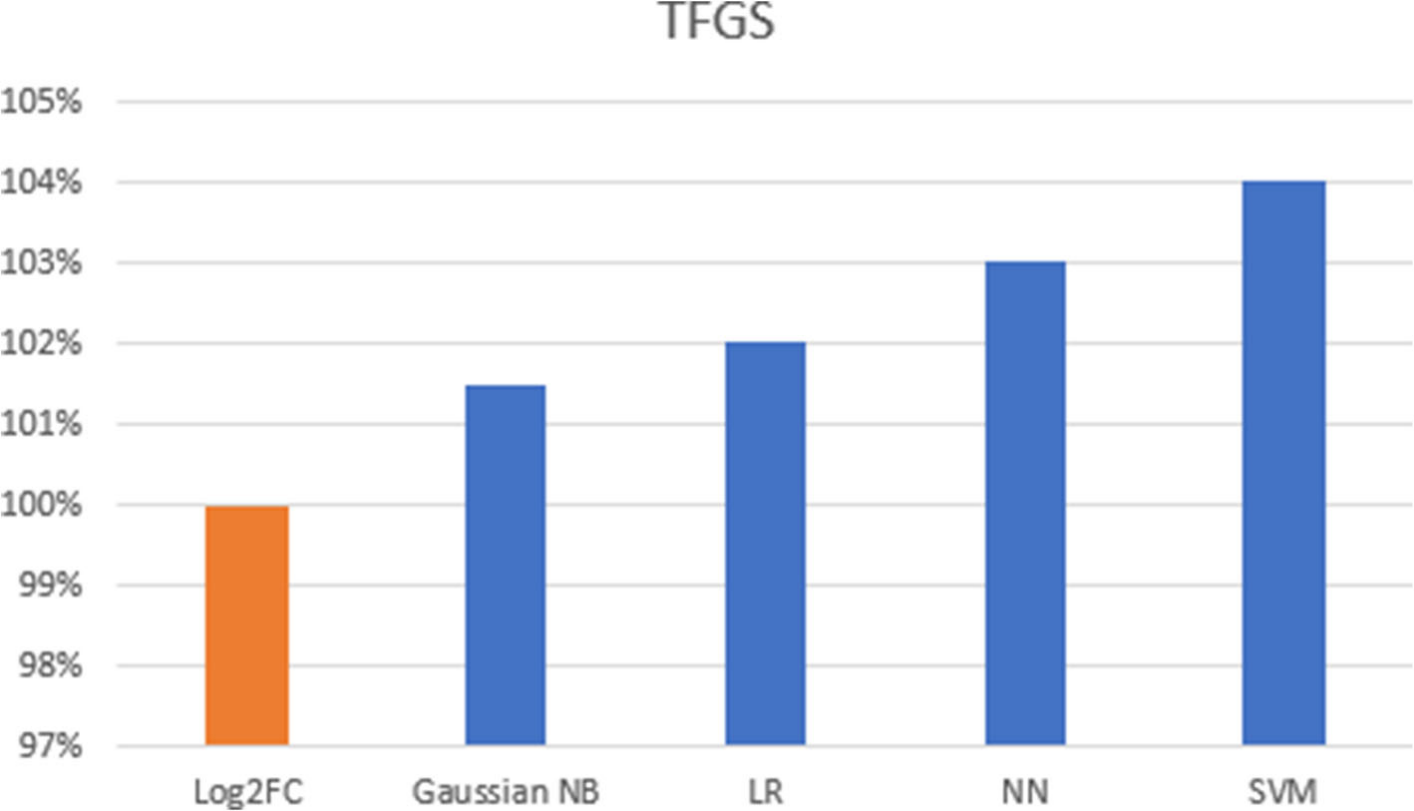
TFGS gained by each machine learning model compare to it gained by log2 ratio only. The higher score means more FGs in all CPs follow the expectation of activation or inhibition

Another evaluation experiment was performed by using TFGS to check if the number of FGs follows the expectation or not. The results are summarized in Fig. 8. TFGS was calculated based on the gene in FGs by replacing its log2 ratio with the predicted results (-1 means down-regulated and 1 means up-regulated). Since there are 14 FGs related to osteoclast Up/Down context, all models are trained 14 times. Each time, one FG was held out as a test set and 13 other FGs were used for the training set. This “cross-validation” like method produced 14 FGSs which were used to calculate TFGS based on the number of FGSs whose regulation direction follows the expectation. TFGS produced by four models were compared to the TFGS produced by using log2FC. In summary, we find that the NN model had the highest AUC area when using the ROC curves. But the SVM model produces the highest TFGS and the highest specificity compared to the rest of the models. We performed the regulatory pattern prediction using all four learned models. Results obtained from these models are then compared to the prediction based on the log2FC only model.

The results of predicting IRF8 and NFATc1 targets are shown in Tables 1 and 2. The error tolerance parameter *T* was set to 5% in this comparison study. Table 7 and Table 8 show the target genes identified differently between the two machine learning models. For clarification we also show the actual feature values for the differentially identified genes and the class label (i.e., GA or GP) that the learned model assigned for each gene in Table 9. BID in CP IDs 5 and 6 has positive log2 ratios, but both CPs have negative row-wise Z-scores in both control and test populations. Both SVM and NN predicted that these two genes are GA. CCRL2 in CP ID 9 has a negative log2 ratio, but all of its Z-scores are positive. Thus, SVM and NN could have classified that this is a GP case. We note that SVM and NN produce the same prediction for most of the genes, but there are cases in which the two models disagree: TAP2 in CP ID 10 and CASP1 in CP ID 16. We conjecture that such disagreement suggests a possible future study to develop an ensemble method capable of combining decisions from multiple learned models.

**Table 7:** Distinct target genes of IRF8 in SVM and NN models compare to log2FC

Models	Differently called genes
SVM	PARP8; BID; CCRL2; CTPS2; CASP1; H2-M3; SLC15A3; IRF5
NN	TAP2; BID; BST2; CCRL2; CTPS2; H2-M3; NOTCH1

**Table 8:** Distinct target genes of NFATc1 in SVM and NN models compare to log2FC

Models	Differently called genes
SVM	ACAD10; CNIH4; DNAJA3; DUS3L; FEM1A; PEX16; PIGQ; PRDX3; SEMA7A; SSNA1; ANXA6; ATF3; CCL3; CDC42EP3; CPEB2; DCK; IRF5; LXN; MAP4K2; PIK3CG; SLC15A3; SLC9A3R1; SP100; TEX2
NN	ACAD10; COG8; COX17; DNAJA3; DUS3L; HINT2; MBTPS1; MRPL12; PRDX3; PTDSS2; TCF12; UMPS; ADAM15; CCL3; CPEB2; LXN; MAP4K2; MS4A7; NAB2; NOTCH1; SLC15A3; SLC9A3R1; SNAP29; TEX2; TMBIM1

**Table 9:** Targt genes with 5 features in different CP and their prediction results

CP ID	Gene Symbol	F1	F2	F3	F4	F5	SVM prediction result	NN prediction result	Pattern
9	NOTCH1	–	+	+	–	–	Up	Up	GP
5	BID	+	+	+	–	–	Down	Down	GA
6	BID	+	+	+	–	–	Down	Down	GA
5	IRF5	+	+	+	+	–	Down	Down	GA
9	CCRL2	–	+	+	+	+	Up	Up	GP
5	CASP1	+	+	+	+	–	Down	Down	GA
16	CASP1	+	+	+	+	+	Down	UP	-
10	TAP2	–	+	+	+	+	Up	Down	-
6	CTPS2	+	+	+	+	–	Down	Down	GA

### Conclusions

IRF8 and NFATc1 are two important transcription factors known for osteoclast differentiation. Although, their potential targets have been widely studied, the regulation patterns of their target genes during osteoclastogenesis are still unknown. By designing and implementing cTAP we identified putative targets of IRF8 and NFATc1 that may play key roles for osteoclast differentiation. The literature survey we performed implicated all the ML method predicted genes in the proper context, i.e., bone remodeling or osteoclast differentiation under which this TF target prediction was performed, providing confidence on the predictive power of cTAP.

Regarding the specifics of cTAP’s predictive power in our study, we report that when cross-referencing the functional group genes summarized in Table 5 with the putative target genes summarized in Tables 1 and 2, two sets of matching genes emerged: for IRF8, MARCKS (“4. Cytoskeleton Control”), and CCL6 (“9. Autoregulatory-down”, and “14. Secreted Factors for External Cells-down”); and for NFATc1, SDC1 (“1. Autoregulatory-up”), TRAF1 (“2. Cell Differentiation signaling factors-down”), MARCKS (“4. Cytoskeleton Control”), SEMA7A (“7. Coupling Factors”), ATP6VOB (“8. ACID & Enzymes for Matrix Dissolution”), CCL3 (“9. Autoregulatory-down”, and “14. Secreted Factors for External Cells-down”). Since the predicted targets were chosen from ENCODE identified putative ChIP targets, one can say that these matching genes could be direct targets of IRF8 and NFATc1, respectively, for the annotated functional groups. These specific relationships are new findings for the scientists who study osteoclastogenesis, and these new discoveries could help bone biologists design validation experiments. For example, to test MARCKS is indeed a direct downstream target of either IRF8 or NFATc1, one can design and perform an IRF8 knock out (KO) study and measure how much cytoskeleton control function is impacted through down/up regulation of MARCKS. Similar hypothesis can be attempted, that is, if knocking out IRF8 diminishes the expression level of CCL6 and at the same time noticeable impact is observed among secreted factors for external cells.

One unique aspect of developing cTAP includes the introduction of gene expression values themselves, i.e., z-scores, in the analysis. Doing so was motivated by recognizing that profiling a gene’s expression simply based on fold change between test and control sample does not utilize all of the information present in gene expression studies. Experimentalists may argue that despite some gene transcripts being expressed at a lower level in the test sample compared to the control sample, their level of expression may still be sufficient enough to carry out its intended function. Likewise, gene transcripts expressed at a higher fold change in the test sample compared to the control sample may not necessarily indicate an up-regulation even if such a decision of differential expression was obtained with statistical significance after background noise removed. The article by [46] points out that merely using fold-change to determine significant changes in gene expression does not reflect signal intensity and can result in a substantial number of generating false positives and false negatives. The design behind cTAP is that if we take into account the collective behaviors of related genes in a cohort of gene expression data sets of the same or comparable context, this potential problem of false positives and false negatives could be abated. We consider that using machine learning methods for this type of prediction problem is very appropriate because designing an algorithmic solution to combine fold-change and signal intensities together introduces too many degrees of freedom in the analysis.

Regarding future work, cTAP offers new ways to “in-silico” assess predicted targets’ implication in skeletal biology, specifically offering venues to associate predicted targets with prior knowledge known or hypothesized for bone diseases. As shown in Tables 1 and 2, too many genes emerged as potential leads. Next task is to study how to prioritize these so that experimentalists can focus on a smaller list of candidates with higher probability for translational potential to address the disease known for its extreme polygenic nature with estimated thousands of low effect genes [47, 48]. One resource to cross-reference the predicted target genes is the International Mouse Phenotyping Consortium (IMPC) repository which is a remarkable resource built through community efforts to identify genes affecting tissue health (including bone) by knocking out thousands of mouse genes individually. Another resource is Human GWAS Catalog that assembled hundreds of loci that are associated with bone mineral density (BMD), osteoporosis, and osteoporotic fractures [49]. Yet another source is systems biology derived gene networks such as the one developed by Al-Barghouti et al. who performed a systems genetics analysis of 55 complex skeletal phenotypes using cortical bone RNA-seq data and reported 66 likely causal genes for human BMD GWAS associations [50]. The key to this type of secondary “in-silico” meta-analysis is to perform an integrative analysis in the most productive and informative manner with sufficiently sensitive tools so that one can identify which target identifying variation in skeletal metrics is predictive of subsequent skeletal dysfunction.

In summary, we point out that development of cTAP originated from our effort to analyze our high throughput *μ*CT and histomorphometric screen of unselected homozygous gene KO mice produced by the IMPC. The IRF8 KO line had the lowest bone mass and highest osteoclastic activity of the 220 lines examined [51] as the details of this analysis can be seen on the webportal, bonebase.org. Understanding which other genes IRF8 could interact with was discoverable by applying cTAP to multiple publicly available gene expression data sets using a proven pathway known to affect one or more of the cell lineages involved in skeletal formation and maintenance. Our next research agenda is to narrow down the predicted target list and engage in validation of promising targets and eventually demonstrate that cTAP like TF target prediction method can be instrumental to deconvoluting the genetic complexity of developmental and degenerative skeletal disease.

## Data Availability

All data analyzed during this study are included in this article. Data and codes are available at https://github.uconn.edu/how17003/cTAP.
